# Two novel *GAA* missense variants and a known VUS in pompe disease: Functional characterization and pharmacological chaperone therapy

**DOI:** 10.1016/j.gendis.2026.102191

**Published:** 2026-04-17

**Authors:** Yang Xia, Yue Song, Ziyue Peng, Yuhuan Meng, Shihui Yu, Xue Zhang

**Affiliations:** a Heilongjiang Provincial Key Laboratory of Child Development and Genetic Research, Harbin Medical University, Harbin, Heilongjiang 150081, China; b NHC and CAMS Key Laboratory of Molecular Probe and Targeted Theranostics, Harbin Medical University, Harbin, Heilongjiang 150081, China; c Department of Pediatrics, 2nd Affiliated Hospital of Harbin Medical University, Harbin, Heilongjiang 150001, China; d KingMed School of Laboratory Medicine, Guangzhou Medical University, Guangzhou, Guangdong 510005, China

Pompe disease (glycogen storage disease type II) is a rare autosomal recessive disorder caused by glycogen-degrading lysosomal enzyme acid α-glucosidase (GAA) deficiency, leading to pathological glycogen accumulation and multisystem dysfunction, primarily affecting muscles.^1^ *GAA* mutations result in varying residual α-glucosidase activity and are closely associated with a broad phenotypic spectrum.^2^ Comprehensive characterization and mapping of the *GAA* mutation spectrum are crucial for early diagnosis, treatment and prognosis.

In this study, we reviewed 39 unique nonsynonymous *GAA* variants identified in 42 samples collected between 2017 and 2020. Some individuals were suspected to have Pompe disease based on genetic testing results and clinical phenotype analysis, while others were classified as *GAA* variant carriers (Fig. S1A and Tables S1 and S2). These variants included 24 missense variants, 9 frameshifts, 3 nonsense variants, and 3 splicing variants. We reassessed these genetic variants according to the American College of Medical Genetics and Genomics/Association for Molecular Pathology (ACMG/AMP) guidelines into 3 subcategories: 11 pathogenic variants, 13 likely pathogenic variants, and 15 variants of uncertain significance (VUS) (Fig. S1B). We further systematically mapped the *GAA* mutation spectrum in our cohort, including the distribution of variant types and their localization to conserved amino acid residues (Fig. S1C). Of the total missense variants detected, we selected three variants—c.521A>G (p.E174G), c.1381G>T (p.G461C), and c.1861T>G (p.W621G)—for detailed analysis based on their localization to highly conserved residues and potential functional impact. Among these, c.521A>G has been previously annotated as a VUS in ClinVar (accession: VCV004525899.1) and reported in a prior study,^3^ whereas c.1381G>T and c.1861T>G are novel variants with no previous records in databases or literature. All three variants were predicted to be deleterious by multiple *in silico* tools, with no splicing effects identified (Tables S3 and S5). Their locations in evolutionarily conserved residues are further illustrated in Figure S1D. Furthermore, VarSite analysis revealed significant hydrophobicity changes for all three variants (Fig. S1E). Structural modeling (SWISS-MODEL) showed reduced hydrogen bonds in p.E174G and p.G461C (Fig. S1F). AlphaMissense classified all three missense variants as ambiguous/likely pathogenic, with nine stability prediction tools consistently indicating destabilizing effects (Table S4). Collectively, these *in silico* findings provide strong supportive evidence for the potential pathogenicity of the three variants.

To further characterize these variants functionally, we transfected COS-7, HeLa, CHO–K1, and HEK293 cells with four *GAA* vectors: three missense variants (p.E174G, p.G461C, p.W621G) and one known pathogenic control (p.E888∗). Endogenous GAA activity was minimal in all cell lines compared to exogenous wild-type (WT) GAA. Exogenous mutant GAA showed significantly reduced activity relative to WT GAA (Fig. 1A–D). Interestingly, our analysis discovered that p.G461C and p.W621G maintained enzyme activity levels comparable to endogenous GAA, whereas p.E174G exhibited significantly higher activity than other mutants (Fig. 1E–H). We performed Western blotting analysis using lysates from four cell lines transfected with either WT or mutant GAA. The 110-kDa precursor, 95-kDa intermediate, and 76-kDa mature forms were detected in WT GAA-expressing cells. While the precursor levels of the three missense variants were comparable to WT GAA, their intermediate and mature forms were barely detectable. These findings underscore the essential role of proteolytic processing in regulating GAA enzymatic function (Fig. 1I–L).

**Figure 1:**
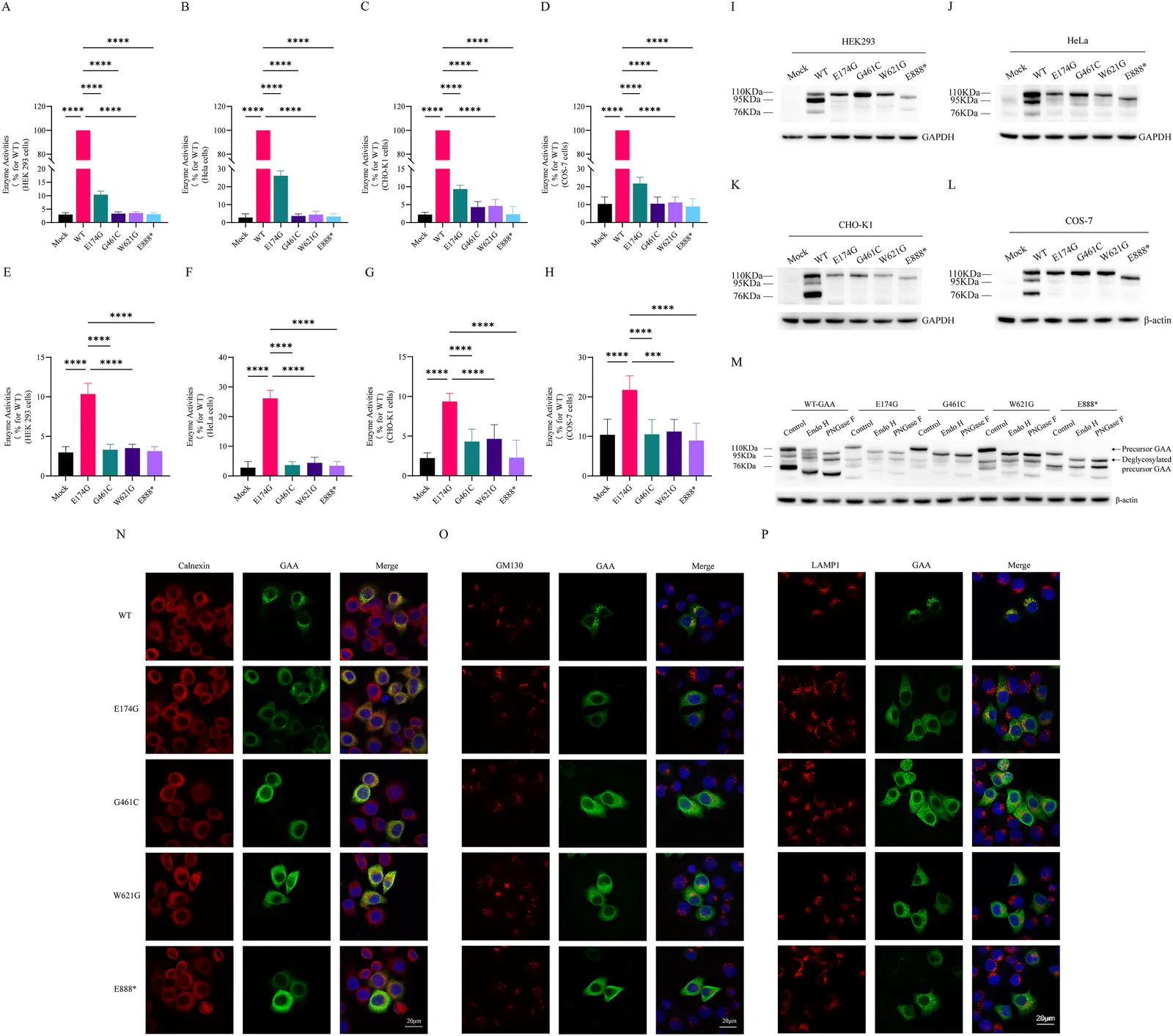
GAA mutants do not undergo proteolytic cleavage and accumulate in the endoplasmic reticulum (ER). **(A**–**H)** Enzyme activities of exogenous expression of WT or mutant GAA in different cell lines. The enzyme activities of all four mutants were low compared with those of WT GAA. The enzyme activities of E174G were significantly higher than those of the other three variants (mean ± SD, *n* ≥ 3, Student's *t*-test, ∗∗∗∗*p* < 0.0001) **(I**–**L)** Western blotting analysis of WT or mutant GAA exogenously expressed in different cell lines **(M)** Western blotting analysis of WT or mutant GAA exogenously expressed in HEK293 cells. Cell lysates were treated with PNGase F and Endo H for 1 h each before analysis **(N–P)** Immunofluorescence staining analysis of WT and mutant GAA, calnexin, GM130, and LAMP1 in HeLa cells. Red fluorescence for calnexin (N), GM130 (O) or cis-Golgi apparatus (P), and blue fluorescence for the nuclei.

Fully active acid α-glucosidase requires sequential post-translational modifications in the endoplasmic reticulum (ER), Golgi apparatus, and endosomes/lysosomes.^4^ We analyzed GAA glycosylation in transiently transfected HEK293 cells using Endo H (cleaves high-mannose N-glycans) and PNGase F (cleaves all N-glycans) (Fig. 1M). Besides little standard molecular weight intermediate forms retained after treatment with Endo H, the precursor forms, intermediate, and mature forms of WT GAA have lower molecular weights after being treated with PNGase F and Endo H, respectively, with partial Endo H-resistant intermediates suggesting trans-Golgi carbohydrate modifications. The mutant proteins only exhibited deglycosylated precursors, indicating impaired trans-Golgi processing (Fig. 1M).

To determine the subcellular localization of the mutant proteins and assess their lysosomal transport, we performed immunofluorescence staining on HeLa cells expressing exogenous WT or mutant GAA. Compared to WT GAA, the mutant proteins showed significantly higher colocalization with the ER marker calnexin (Fig. 1N). None of the four mutants colocalized with the cis-Golgi marker GM130 (Fig. 1O), and lysosomal delivery was markedly impaired (Fig. 1P).

Based on previous studies,^5^ we speculated that endoplasmic reticulum-associated degradation (ERAD) may degrade these mutant GAA and that the suppression of autophagy may be beneficial for mutant GAA. To address the possibility, HeLa cells and HEK293 cells expressing with WT or mutant GAA were treated with MG132 (an inhibitor of proteasome-mediated degradation) and bafilomycin A1 (Baf) (an inhibitor of lysosome-mediated degradation), respectively. Notably, WT GAA exhibited high stability with only minor autophagy-dependent degradation (Fig. S2A and S2F). In contrast, mutant GAA was markedly unstable and predominantly degraded via ERAD (Fig. S2B–S2E, S2G–S2J).

Small molecule pharmacological chaperones have been considered as a potential alternative therapy for Pompe disease, independent or complementary to enzyme replacement therapy (ERT). These compounds could specifically bind to and stabilize enzymes during synthesis in the ER, thus properly facilitating protein folding and trafficking.^1^ To assess the response to imino sugars, WT and mutant GAA was transiently expressed in four cell lines and then treated with different concentrations of deoxynojirimycin (DNJ) and N-butyl-deoxynojirimycin (NB-DNJ), respectively. There were no apparent changes in enzyme activities in HeLa cells and HEK293 cells overexpressing mutant GAA after being treated with DNJ or NB-DNJ (Fig. S3E and S3F). The activity of mutant GAA in CHO–K1 cells was significantly improved after being treated with DNJ and NB-DNJ (Fig. S3B), but treatment with DNJ or NB-DNJ did not affect the proteolytic cleavage of mutant GAA in CHO–K1 cells (Fig. S3G). In COS-7 cells, the activity of mutant GAA was significantly improved after being treated with DNJ and NB-DNJ (Fig. S3C), and after treatment with 50 μM NB-DNJ, the translocation of p.E174G to lysosomes was restored (Fig. S3D).

In conclusion, we functionally characterized three missense variants in *GAA*, two of which are novel, expanding the *GAA* mutation spectrum. *In vitro* experiments demonstrated their deleterious impact by showing decreased protein levels and enzyme activities. We further demonstrated that the mutant GAA was prone to remain in the ER and was degraded mainly by ERAD. All three variants responding to DNJ and/or NB-DNJ provided strong *in vitro* evidence for facilitating the treatment of Pompe disease with small molecule pharmacological chaperones.

## CRediT authorship contribution statement

**Yang Xia:** Writing – review & editing, Writing – original draft, Visualization, Software, Methodology, Investigation, Formal analysis, Data curation, Conceptualization. **Yue Song:** Validation, Investigation, Conceptualization. **Ziyue Peng:** Validation, Investigation, Conceptualization. **Yuhuan Meng:** Methodology, Data curation. **Shihui Yu:** Writing – review & editing, Writing – original draft, Methodology, Investigation, Funding acquisition, Conceptualization. **Xue Zhang:** Writing – review & editing, Writing – original draft, Methodology, Investigation, Funding acquisition, Conceptualization.

## Ethics declaration

Approved by the Ethics Committee of Harbin Medical University (HMUIRB2022006) in accordance with the Helsinki Declaration, this retrospective study used anonymized variant data, waiving informed consent requirements.

## Data availability

Data are contained within the article and Supplementary material.

## Funding

This research was supported by the 10.13039/501100012166National Key R&D Program of China (No. 2022YFC27039002024YFC3406700).

## Conflict of interests

The authors declare that they have no conflict of interests.
