# Supplementary material for: Two novel GAA missense variants and a known VUS in pompe disease: Functional characterization and pharmacological chaperone therapy
Source: Genes Dis. 2026 Apr 17;14(2):102191. doi: 10.1016/j.gendis.2026.102191 (PMC13634567; doi:10.1016/j.gendis.2026.102191)
Supplement: Supplementary file 1 [file mmc1.docx]

Materials and Methods

Samples

The 42 samples carrying *GAA* variants were previously collected in our database. Some individuals were suspected of Pompe disease patients based on genetic testing results and clinical phenotype analysis, while the others were considered GAA variant carriers. Detailed information about these samples is summarized in Table S1.

Variants Calling, Annotation, and Interpretation

In the current study, *GAA* variants and relevant information from these samples were retrospectively reviewed and systematically re-analyzed (Tables S1 and S2). In brief, these samples' genetic/genomic test results were performed on the Illumina NexSeq 500 or HiSeq 2500 sequencing platforms. Genetic variants were annotated based on the human genome Reference sequences NM_000152.5 and LRG_673t1.1. The clinical significance of genetic/genomic variants was assessed based on the guidelines from the American College of Medical Genetics and Genomics and the Association for Molecular Pathology (ACMG/AMP). All of the annotated variants, including SNVs, indels, and CNVs, were classified into 5 categories based on the evaluation of their clinical significances: (1) pathogenic variants; (2) likely pathogenic variants; (3) variants of uncertain significance (VUS); (4) likely benign variants; and (5) benign variants. Sanger sequencing was performed on the ABI-3730xl system for follow-up testing for the available parents and relatives of the probands.

*In Silico* Pathogenicity Prediction and Three-Dimensional Structural Remodeling

We used 7 scoring standards based on *in silico* tools and databases to comprehensively assess the pathogenicity of 3 missense variants (Table S3). Additionally, 5 *in silico* tools were used to assess the mis-splicing of three missense variants (Table S5). VarSite was used to predict the changes in amino acid hydrophobicity at the 3 missense variants. SWISS-MODEL was used to predict the three-dimensional (3D) structures of wild-type (WT) and mutant forms of GAA. Ten tools were used to predict pathogenicity and protein stability (Table S4). PyMOL 2.6.0 was used for the 3D structure prediction and visualization.

Cell Culture

HeLa cells, HEK293 cells, COS-7 cells, and CHO-K1 cells were purchased from the National Collection of Authenticated Cell Cultures, China. HeLa cells, HEK293 cells and COS-7 cells are grown in Dulbecco’s modified Eagle’s medium (DMEM) (Gibco, Rockville, MD, USA) supplemented with 10% fetal bovine serum (FBS) (Sigma-Aldrich, St Louis, MO) with 100x GlutaMAX (Gibco) and CHO-K1 cells are grown in Ham's F-12K (Kaighn's) medium (Gibco) supplemented with 10% fetal bovine serum (FBS) (Sigma-Aldrich) with 100x GlutaMAX (Gibco). All the cells are maintained at 37 °C in a 5% CO2, 95% humidified air atmosphere.

Plasmid Constructions, Transfection, and Treatment

pCMV-sport6-GAA plasmid containing GAA wild-type (WT) cDNA (BC010210.1) was purchased from Miaoling Biology, China. Site-directed mutagenesis was used to obtain GAA WT reference sequences cDNA (NM_000152.5). GAA cDNA was introduced into the pcDNA3.1(+) and pIRES2-EGFP expression vectors, respectively. Then, site-directed mutagenesis was used to obtain mutations for variants c.521A>G(p.E174G), c.1381G>T(p.G461C), c.1861T>G(p.W621G), and c.2662G>T(p.E888*) based on the WT cDNA reference sequence (NM_000152.5). Site-directed mutants were transfected in HeLa cells, HEK293 cells, COS-7 cells, and CHO-K1 cells and cultured for 24 h to 48 h, with transient transfection reagent Effectene (Qiagen, Valencia, CA) or Lipofectamine (Invitrogen, Carlsbad, CA, USA), according to the manufacturer’s instructions. For cell treatments, 10 μM or 50 μM MG132 (Selleck, Houston, TX, USA and MedChemExpress, New Jersey, USA), 400 nM bafilomycin A1 (MedChemExpress), 20 μM or 50 μM DNJ (Cayman Chemical, Michigan, USA) or NB-DNJ (MedChemExpress) were applied.

Protein Preparation and Western Blotting

Proteins were extracted from HeLa cells, HEK293 cells, COS-7 cells, and CHO-K1 cells in RIPA lysis buffer (Thermo Fisher Scientific, Waltham, MA, USA) with the protease inhibitor cocktail (Selleck) at 4℃ or a cell lysis buffer containing 10mM NaF, 1mM Na3VO4, 0.5% SDS and 1X PBS at room temperature. The samples were subjected to SDS-PAGE followed by western blotting procedures. The following antibodies were used for immunoblotting: anti-β-actin (Proteintech, Wuhan, China), anti-GAPDH (Proteintech), anti-EGFP (Proteintech), and anti-GAA (Invitrogen). For digestion of carbohydrates, PNGase F (N-glycosidase F) (New England Biolabs, Ipswich, MA, USA) and Endo H (endoglycosidase H) (New England Biolabs) were mixed with cell lysates at 37 °C for 1 h in Glycobuffer (New England Biolabs) and 1% NP40 (New England Biolabs).

Immunofluorescence Staining

HeLa and COS-7 cells were grown on coverslips and fixed in paraformaldehyde, followed by permeation in Methanol, blocking with 5% house serum albumin. Immunofluorescence staining was performed using the following antibodies: anti-GAA (Invitrogen), anti-LAMP1 (Cell Signaling, Danvers, MA, USA), anti-calnexin (Cell Signaling), anti-GM130 (Cell Signaling). Cells were visualized under a Zeiss confocal microscope (Carl Zeiss, Jena, Germany). The merge ratios for describing the immunoreactivities of GAA with calnexin, GM130, or LAMP1 were measured using ImageJ (National Institutes of Health, Rockville, MD, USA).

Measurement of Enzyme Activities

The activities of the GAA proteins produced by the transfected plasmids were measured using 4-methylumbelliferyl-α-D-glucopyranoside (4-MUG) (Cayman Chemical) as a substrate in transfected cells. Reaction mixtures contained 5μg protein in 0.1 M sodium acetate (pH 4.8), being applied for 1 h at 37 ℃ in a dark room and stopped by adding 200μl of 1 M glycine–NaOH (pH 10.5). The released 4-methylumbelliferone (4-MU) fluorescence was measured using a BioTek Synergy H1 multimode reader, with excitation and emission wavelengths of 360 nm and 465 nm, respectively. Reactions were set up in duplicates, and measurements were corrected for background emission from reactions containing no enzyme. Data were analyzed and graphed with Prism (GraphPad Prism version 9.4.0 for Windows; GraphPad Software, www.graphpad.com).

Statistical Analysis

Statistical comparisons were made using an unpaired Student’s t-test (between two samples) and analysis of variance (ANOVA) followed by the Bonferroni post hoc test (among multiple samples). All error bars were S.E.M (Standard Error of the Mean). GraphPad Prism 9.4.0 was used to determine the statistical significance, F (F-statistic), and t (T-statistic) values. The statistical significance of a difference was determined based on a *P* < 0.05. *P*-values <0.05, 0.01, 0.001, or 0.0001 were presented as **P* < 0.05, ***P* < 0.01, ****P* < 0.001, or *****P* < 0.0001, respectively.

**Figure S1. Variants analysis.** (**A**) Gene variant count and Types. (**B**). The American College of Medical Genetics and Genomics and the Association for Molecular Pathology (ACMG/AMP) pathogenicity classification of *GAA*. (**C**) Gene mutation spectrum of *GAA*. (**D**). Evolutionary conservation of amino acids of 3 *GAA* missense variants. (**E**) Prediction of the amino acid hydrophobicity. (**F**) Three-dimensional modeling of GAA structure.

**Figure S2. Mutant GAA are unstable and are degraded by the ERAD pathway and autophagy.** (A-E) Western blotting analysis of GAA in HEK293 cells expressing WT and mutant GAA. (F-J) Western blotting analysis of GAA in HeLa cells expressing WT and mutant GAA (N=3). Cells were treated with 10 µM MG132 or 400 nM Baf for 6 hours. The treatment with MG132 significantly increased the amounts of the mutant GAA, and cotreatment of MG132 and Baf dramatically increased the amounts of the precursor forms of mutant GAA expect for G461C. MG132: proteasome inhibitor; Baf: bafilomycin A1, inhibiting lysosomal acidification by the vacuolar H+-ATPase.

**Figure S3. Rescue of mutant GAA enzyme activity by DNJ/NB-DNJ treatment.** (A) The chemical structure of DNJ (left) and NB-DNJ (right). (B-C) Increased enzymatic activity of mutant GAA in DNJ/NB-DNJ-treated CHO-K1 and COS-7 cells. Cells transiently expressing mutant GAA were treated with 0, 20, or 50 μM DNJ or NB-DNJ (N=3). Data represent mean ± SD. Compared with the absence of DNJ and NB-DNJ, obvious improvements after treatment with DNJ or NB-DNJ were obtained. (**P* <0.05, ** *P* <0.005, *** *P* <0.001, *****P* < 0.0001). (D) Lysosomal localization of mutant GAA in COS-7 cells exogenously expressing WT and mutant GAA was observed after treatment with DNJ or NB-DNJ. Cells were labeled with antibodies to GAA (green) and LAMP2 (red). In COS-7 cells expressing mutant GAA in the absence of DNJ or NB-DNJ, negligible immunofluorescent labeling of lysosomal localization of mutant GAA was observed. NB-DNJ treatment of the cells expressing E174G resulted in an enhancement of immunofluorescent labeling of lysosomal localization of GAA, which pattern was comparable to that observed in the cells expressing the WT GAA. (E-F) Enzyme activities of exogenous expression of mutant GAA in HEK293 cells and HeLa cells in the absence and presence of DNJ or NB-DNJ. (E) HKE293 cells; (F) HeLa cells. There was no significant alteration of enzyme activities of mutant GAA observed in the presence of DNJ or NB-DNJ. (G) Western blotting analysis of WT and mutant GAA exogenous expressing in CHO-K1 cells incubated with 0, 20, or 50 μM DNJ or NB-DNJ were obtained.

Table S1. Clinical information and mutation spectrum of the *GAA* in 42 Chinese samples.

| NO. | Gender | Age | Symptoms or signs | Variants | Evidence of pathogenicity* | References (PMID) |
| --- | --- | --- | --- | --- | --- | --- |
| 1 | F | 29y | NA | c.503G>A (p. Arg168Gln) | VUS (PM2_supporting, PP2) | 21488292 |
| 2 | F | 12y | NA | c.521A>G (p. Glu174Gly) | VUS (PM2_supporting, PP3) | 38186848 |
| 3 | F | 23y | NA | c.671G>A (p. Arg224Gln) | Likely pathogenic (PS3_supporting, PM2_supporting, PM3, PM5, PP3) | 21488293 |
| 4 | M | 35y | NA | c.796C>T (p. Pro266Ser) | Likely pathogenic (PS3_supporting, PM2_supporting, PM3_strong) | 17092519 |
| 5 | M | 6d | NA | c.953T>A (p. Met318Lys) ^b^ | VUS (PM1, PM2_supporting, PM3_supporting, PP3) | 18425781 |
| 6 | F | 5m | 1 | c.1082C>T (p. Pro361Leu) | Pathogenic (PS3_supporting, PM2_supporting, PM3_very strong, PP3) | 12601120 |
| 7 | M | 25y | NA | c.1210G>A (p. Asp404Asn) | Likely pathogenic (PM2_supporting, PM3_strong, PP3) | 16433701 |
| 8 | M | 25y | 4,5,6,7 | c.1562A>T (p. Glu521Val) | VUS (PM2_supporting, PM3_supporting, PP3) | 25526786 |
| 9 | NA | NA | NA | c.1735G>A (p. Glu579Lys) | Likely pathogenic (PS3_supporting, PM2_supporting, PM3_strong, PP3) | 14695532 |
| 10 | M | 20y | NA | c.1843G>A (p. Gly615Arg) | Likely pathogenic (PM1, PM2_supporting, PM3_strong, PP3, PP4) | 10338092 |
| 11 | M | 3y | NA | c.2173C>T (p. Arg725Trp) ^a^ | Likely pathogenic (PS3_supporting, PM2_supporting, PM3, PP3, PP4) | 8401535 |
| 12 | F | 53y | 2 | c.2236T>G (p. Trp746Gly) | Likely pathogenic (PS3_supporting, PM2_supporting, PM3, PP3, PP4) | 20080426 |
| 13 | M | 61y | 2,3 | c.2237G>A (p. Trp746*) | Pathogenic (PVS1, PM2_supporting, PM3_very strong, PP4) | 24158270 |
| 14 | M | 28y | NA | c.2662G>T (p. Glu888*) | Pathogenic (PVS1, PM2_supporting, PM3, PP4) | 17723315 |
| 15 | M | 30y | NA | c.2662G>T (p. Glu888*) | Pathogenic (PVS1, PM2_supporting, PM3, PP4) | 17723315 |
| 16 | M | 6y | 2,3 | c.252dupC (p. Asn87Gln) ^a^ | Pathogenic (PVS1, PM2_supporting, PM3_supporting) | 10206684 |
| 17 | NA | NA | 2,3 | c.525_526del (p. Asn177Pro) | Pathogenic (PVS1, PM2_supporting, PP4) | 22252923 |
| 18 | M | 6.6m | NA | c.735delC (p. Leu246Phe) | Likely pathogenic (PVS1, PM2_supporting) | 18425781 |
| 19 | F | 3m | NA | c.1355delC (p. Ser454Ala) | Pathogenic (PVS1, PM2_supporting, PP4) | 22252923 |
| 20 | F | 58y | 8,9 | c.1411_1414del (p. Glu471Pro) | Pathogenic (PVS1, PM2_supporting, PM3) | 8604985 |
| 21 | F | 28y | NA | c.546+5G>T | VUS (PS3_supporting, PM2_supporting, PP3) | 20080426 |
| 22 | M | 29y | NA | c.546+5G>T | VUS (PS3_supporting, PM2_supporting, PP3) | 20080426 |
| 23 | NA | NA | NA | c.859-2A>T | Pathogenic (PVS1, PM2_supporting, PM3_supporting) | 24269976 |
| 24 | F | 2.5y | 10 | c.460C>T (p. Arg154Cys) | VUS (PM2_supporting, PM5_supporting, PP3) | - |
|  |  |  |  | c.503G>A (p. Arg168Gln) | VUS (PM2_supporting, PP2) | - |
| 25 | M | 16y | PD,12 | c.521A>G (p. Glu174Gly) ^a^ | VUS (PM2_supporting, PP3) | 38186848 |
|  |  |  |  | c.521A>G (p. Glu174Gly) ^b^ | VUS (PM2_supporting, PP3) |  |
| 26 | F | 17y | 2,3 | c.752C>T (p. Ser251Leu) | VUS (PS3_supporting, PM2_supporting, PM3_supporting) | -- |
|  |  |  |  | c.761C>T (p. Ser254Leu) | VUS (PP3) |  |
| 27 | F | 31y | NA | c.-32-13T>G | Likely pathogenic (PM2_supporting, PM3_very strong) | 7881425 |
|  |  |  |  | c.796C>T (p. Pro266Ser) | Likely pathogenic (PS3_supporting, PM2_supporting, PM3_strong) | 17092519 |
|  |  |  |  | c.1411_1414del (p. Glu471Pro) | Pathogenic (PVS1, PM2_supporting, PM3) | 8604985 |
|  |  |  |  | c.2238G>C (p. Trp746Cys) | Pathogenic (PM2_supporting, PM3_very strong, PM5, PP3, PP4) | 18458862 |
| 28 | M | 4y | NA | c.1082C>T (p. Pro361Leu) | Pathogenic (PS3_supporting, PM2_supporting, PM3_very strong, PP3) | 12601120 |
|  |  |  |  | c.2051C>T (p. Pro684Leu) | VUS (PM2_supporting, PP3) | - |
| 29 | M | 1.67y | 1,2,6 | c.1082C>T (p. Pro361Leu) ^a^ | Pathogenic (PS3_supporting, PM2_supporting, PM3_very strong, PP3) | 12601120 |
|  |  |  |  | c.1935C>A (p. Asp645Glu) ^b^ | Likely pathogenic (PS3_supporting, PM3_strong, PP3, PP4) | 8094613 |
| 30 | M | 35y | NA | c.1316T>A (p. Met439Lys) ^b^ | Likely pathogenic (PM2_supporting, PM3_strong, PP3) | 17092519 |
|  |  |  |  | c.2238G>C (p. Trp746Cys) ^a^ | Pathogenic (PM2_supporting, PM3_very strong, PM5, PP3, PP4) | 18458862 |
| 31 | F | 6m | 1 | c.1381G>T (p. Gly461Cys) ^b^ | VUS (PM2_supporting, PP3) | novel |
|  |  |  |  | c.2662G>T (p. Glu888*) ^a^ | Pathogenic (PVS1, PM2_supporting, PM3, PP4) | 17723315 |
| 32 | M | 3y | NA | c.1669A>T (p. Ile557Phe) ^a^ | VUS (PM1, PM2_supporting, PM3_supporting, PP3) | 20080426 |
|  |  |  |  | c.2132_2133delinsGG (p. Thr711Arg) ^a^ | VUS (PM2_supporting, PP3, PP4) | 18211760 |
| 33 | F | 15y | NA | c.2184delC (p. Leu729Trp) ^a^ | Pathogenic (PVS1, PM2_supporting, PM3) | 28394184 |
|  |  |  |  | c.2238G>C (p. Trp746Cys) | Pathogenic (PM2_supporting, PM3_very strong, PM5, PP3, PP4) | 18458862 |
|  |  |  |  | c.1726G>A (p. Gly576Ser) | VUS (PM5, PP2, PP3, BA1) | 14643388 |
| 34 | F | 5d | 1,13,14 | c.1802C>T (p. Ser601Leu) ^a^ | Likely pathogenic (PM1, PM2_supporting, PM3_strong, PP3, PP4) | 22252923 |
|  |  |  |  | c.1822C>T (p. Arg608*) ^b^ | Pathogenic (PVS1, PM2_supporting, PM3, PP4) | 20202878 |
| 35 | F | 8.8m | 1,2,3 | c.1843G>A (p. Gly615Arg) ^a^ | Likely pathogenic (PM1, PM2_supporting, PM3_strong, PP3, PP4) | 10338092 |
|  |  |  |  | c.2184delC (p. Leu729Trp) ^b^ | Pathogenic (PVS1, PM2_supporting, PM3) | 28394184 |
| 36 | M | 31y | NA | c.1843G>A (p. Gly615Arg) | Likely pathogenic (PM1, PM2_supporting, PM3_strong, PP3, PP4) | 10338092 |
|  |  |  |  | c.2238G>C (p. Trp746Cys) | Pathogenic (PM2_supporting, PM3_very strong, PM5, PP3, PP4) | 18458862 |
| 37 | F | 25d | 1 | c.859-2A>T ^a^ | Pathogenic (PVS1, PM2_supporting, PM3_supporting) | 24269976 |
|  |  |  |  | c.1861T>G (p. Trp621Gly) ^b^ | VUS (PM1, PM2_supporting, PP3) | Novel |
| 38 | F | 27y | NA | c.1935C>A (p. Asp645Glu) | Likely pathogenic (PS3_supporting, PM3_strong, PP3, PP4) | 8094613 |
|  |  |  |  | c.2238G>C (p. Trp746Cys) | Pathogenic (PM2_supporting, PM3_very strong, PM5, PP3, PP4) | 18458862 |
| 39 | M | 6m | 1 | c.1411_1414del (p. Glu471Pro) | Pathogenic (PVS1, PM2_supporting, PM3) | 8604985 |
|  |  |  |  | c.1935C>A (p. Asp645Glu) | Likely pathogenic (PS3_supporting, PM3_strong, PP3, PP4) | 8094613 |
| 40 | M | 7d | NA | c.1935C>A (p. Asp645Glu) ^a^ | Likely pathogenic (PS3_supporting, PM3_strong, PP3, PP4) | 8094613 |
|  |  |  |  | c.1935C>A (p. Asp645Glu) ^b^ | Likely pathogenic (PS3_supporting, PM3_strong, PP3, PP4) |  |
| 41 | F | 28y | PD,1,11 | c.2021_2023del (p. N675del) | Likely pathogenic (PM2_supporting, PM3_strong, PM4, PP3) | 9554747 |
|  |  |  |  | c.2238G>C (p. Trp746Cys) | Pathogenic (PM2_supporting, PM3_very strong, PM5, PP3, PP4) | 18458862 |
| 42 | F | 24y | 15 | c.457_462del (p.R154_T155del) ^a^ | Likely pathogenic (PM1, PM2_supporting, PM4, PP5) | 22252923 |
|  |  |  |  | c.2238G>C (p. Trp746Cys) ^b^ | Pathogenic (PM2_supporting, PM3_very strong, PM5, PP3, PP4) | 18458862 |

NA, not available; a, maternal; b, paternal; F, female; M, male; y, year; m, month; d, day; *ACMG/AMP evidence codes applied; PD, Pompe disease; 1, cardiomyopathy; 2, muscle weakness; 3, hypotonia; 4, arthralgia; 5, splenomegaly; 6, hepatic insufficiency; 7, hypoproteinemia; 8, dysphagia; 9, syncope; 10, epilepsy; 11, pneumonia; 12, scoliosis; 13, patent foramen ovale; 14, patent ductus arteriosus; 15, dyspnoea.

Table S2. Types and frequency of *GAA* mutations of 42 samples.

| Nucleotide change | Structural effect | Location | Predicted severity^*^ | Incidence |
| --- | --- | --- | --- | --- |
| Missense mutation | | | | |
| c.460C>T | p. R154C | Exon2 | ^b^ | 1 |
| c.503G>A | p. R168Q | Exon2 | ^b^ | 2 |
| c.521A>G | p. E174G | Exon2 | ^b^ | 2 |
| c.671G>A | p. R224Q | Exon3 | Potentially less severe | 1 |
| c.[752C>T; c.761C>T] | p. [S251L, S254L] | Exon4 | Presumably non-pathogenic | 1 |
| c.796C>T | p. P266S | Exon4 | Potentially mild | 1 |
| c.953T>A | p. M318K | Exon5 | Potentially less severe | 1 |
| c.1082C>T | p. P361L | Exon7 | Potentially less severe | 4 |
| c.1210G>A | p. D404N | Exon8 | Potentially less severe | 1 |
| c.1316T>A | p. M439K | Exon8 | Potentially mild | 1 |
| c.1381G>T^a^ | p. G461C | Exon9 | ^b^ | 1 |
| c.1562A>T | p. E521V | Exon11 | ^b^ | 1 |
| c.1669A>T | p. I557F | Exon12 | Very severe | 1 |
| c.1726G>A | p. G576S | Exon12 | Presumably non-pathogenic | 1 |
| c.1735G>A | p. E579K | Exon12 | Potentially less severe | 1 |
| c.1802C>T | p. S601L | Exon13 | Potentially less severe | 1 |
| c.1843G>A | p. G615R | Exon13 | Potentially less severe | 3 |
| c.1861T>G^a^ | p. W621G | Exon13 | ^b^ | 1 |
| c.1935C>A | p. D645E | Exon14 | Potentially less severe | 5 |
| c.2051C>T | p. P684L | Exon15 | potentially less severe | 1 |
| c.2173C>T | p. R725W | Exon15 | Less severe | 1 |
| c.2132C>G | p. T711R | Exon15 | Non-pathogenic | 1 |
| c.2236T>G | p. W746G | Exon16 | Potentially less severe | 1 |
| c.2238G>C | p. W746C | Exon16 | Potentially mild | 7 |
| nonsense mutation | | | | |
| c.1822C>T | p. R608* | Exon13 | Very severe | 1 |
| c.2237G>A | p. W746* | Exon16 | Very severe | 1 |
| c.2662G>T | p. E888* | Exon19 | Very severe | 3 |
| Deletion or duplication | | | | |
| c.252dupC | p. N87Q | Exon2 | Very severe | 1 |
| c.457_462del | p. R154_T155del | Exon2 | ^b^ | 1 |
| c.525_526del | p. N177P | Exon2 | Very severe | 1 |
| c.735delC | p. L246F | Exon4 | Very severe | 1 |
| c.1355delC | p. S454A | Exon9 | Very severe | 1 |
| c.1411_1414del | p. E471P | Exon9 | Very severe | 3 |
| c.2021_2023del | p. N675del | Exon14 | Potentially less severe | 1 |
| c.2184delC | p. L729W | Exon15 | Very severe | 2 |
| Splicing variant | | | | |
| c.-32-13T>G | - | Intron1 | Potentially mild | 1 |
| c.546+5G>T | - | Intron2 | ^b^ | 2 |
| c.859-2A>T | - | Intron4 | Very severe | 2 |

a, Novel mutation; b, Not described; *, The disease severity of each variant was based on information from Pompe disease *GAA* variant database (https://www.pompevariantdatabase.nl/).

Table S3. Pathogenicity prediction of three missense variants of *GAA* *in silico* predictive tools

| DNA change | Protein change | M.CAP | SIFT | Polyphen2 | Mutation Taster | FATHMM | Mutation-Assessor | LRT |
| --- | --- | --- | --- | --- | --- | --- | --- | --- |
| c.521A>G | p.E174G | Possibly Pathogenic  (0.295) | Damaging | Probably damaging  (0.999) | Disease causing | Tolerated | Disease_causing | Deleterious |
| c.1381G>T | p.G461C | Possibly Pathogenic  (0.423) | Damaging | Probably damaging  (1.000) | Disease causing | Deleterious | Disease_causing | Deleterious |
| c.1861T>G | p.W621G | Possibly Pathogenic  (0.930) | Damaging | Probably damaging  (1.000) | Disease causing | Deleterious | Disease_causing | Deleterious |

Table S4. Pathogenicity and Structural Stability Analysis of three *GAA* missense Mutations.

| DNA change | Protein change | Pathogenicity or structural stability | | | | | | | | | | | | | |
| --- | --- | --- | --- | --- | --- | --- | --- | --- | --- | --- | --- | --- | --- | --- | --- |
|  |  | AlphaMissense | DUET | | | PoPMuSiC | CUPSAT | | DDGun | Mupro | I-Mutant | SAAFEC-SEQ | iStable | Dynamut | |
|  |  |  | mCSM Predicted Stability Change (ΔΔG) | SDM Predicted Stability Change (ΔΔG) | DUET Predicted Stability Change (ΔΔG) |  | Predicted ΔΔG | Torsion* |  | detal delta G: | Stability | ddG | Conf. Score | ΔΔG | ΔΔSVib ENCoM |
| c.521A>G | p.E174G | 0.504  (Ambiguous) | -1.566k  cal/mol  (Destabilizing) | -1.39 kcal/mol  (Destabilizing) | -1.824 kcal/mol  (Destabilizing) | 1.56 kcal/mol  (Destabilizing) | -1.13kcal/mol  (Destabilizing) | Unfavourable | DDG Score: -0.2  (Decrease) | -1.1949677  (DECREASE stability) | Decrease | -0.87  (Destabilizing) | 0.653324  (Decrease) | -1.528 kcal/mol (Destabilizing) | 0.748 kcal.mol-1.K-1 (Increase of molecule flexibility) |
| c.1381G>T | p.G461C | 0.943  (likely_pathogenic) | -0.082 kcal/mol  (Destabilizing) | -0.67 kcal/mol  (Destabilizing) | 0.021 kcal/mol  (Stabilizing) | -0.03kcal/mol  (Stabilizing) | 6.01kcal/mol  (Stabilizing) | Unfavourable | DDG Score: -0.5  (Decrease) | -0.51012249  (DECREASE stability) | Decrease | -1.22  (Destabilizing) | 0.521734  (Decrease) | 1.066 kcal/mol (Stabilizing) | -0.572 kcal.mol-1.K-1 (Decrease of molecule flexibility) |
| c.1861T>G | p.W621G | 0.865  (likely_pathogenic) | -3.142 kcal/mol  (Destabilizing) | -0.77 kcal/mol  (Destabilizing) | -2.679 kcal/mol  (Destabilizing) | 2.89kcal/mol  (Destabilizing) | -2.74kcal/mol  (Destabilizing) | Unfavourable | DDG Score: -4.2  (Decrease) | -1.4741258  (DECREASE stability) | Decrease | -2.34  (Destabilizing) | 0.505213  (Increase) | -2.067 kcal/mol (Destabilizing) | 2.527 kcal.mol-1.K-1 (Increase of molecule flexibility) |

Table S5. Prediction of mis-splicing for three missense variants of *GAA* *in silico* predictive tools.

| DNA change | Protein change | RDCC | EX-skip | dbscSNV | | SpliceAI | Human Splicing Finder |
| --- | --- | --- | --- | --- | --- | --- | --- |
|  |  |  |  | Ada | RF | Δ score |  |
| c.521A>G | p.E174G | Consistent with the WT RNA splicing pattern | Same as WT | - | - | Benign (0.03) | Alteration of auxiliary sequences: Significant alteration of ESE / ESS motifs ratio (-12) |
| c.1381G>T | p.G461C | Consistent with the WT RNA splicing pattern | Same as WT | - | - | Benign (0.02) | No significant impact on splicing signals |
| c.1861T>G | p.W621G | Consistent with the WT RNA splicing pattern | Same as WT | - | - | Benign (0.00) | No significant impact on splicing signals |
